# Genetic defects are common in myopathies with tubular aggregates

**DOI:** 10.1002/acn3.51477

**Published:** 2021-12-15

**Authors:** Qiang Gang, Conceição Bettencourt, Stefen Brady, Janice L. Holton, Estelle G. Healy, John McConville, Patrick J. Morrison, Michela Ripolone, Raffaella Violano, Monica Sciacco, Maurizio Moggio, Marina Mora, Renato Mantegazza, Simona Zanotti, Zhaoxia Wang, Yun Yuan, Wei‐wei Liu, David Beeson, Michael Hanna, Henry Houlden

**Affiliations:** ^1^ 26447 Department of Neurology Peking University First Hospital 8 Xishiku Street, Xicheng District Beijing 100034 China; ^2^ 26447 Beijing Key Laboratory of Neurovascular Disease Discovery Beijing 100034 China; ^3^ 61554 Department of Neuromuscular Diseases UCL Queen Square Institute of Neurology Queen Square London UK; ^4^ 61554 MRC Centre for Neuromuscular Diseases UCL Queen Square Institute of Neurology Queen Square London UK; ^5^ 61554 Queen Square Brain Bank for Neurological Disorders London UK; ^6^ 61554 Department of Neurodegenerative Disease UCL Queen Square Institute of Neurology Queen Square London UK; ^7^ 11269 Oxford Muscle Service John Radcliffe Hospital Oxford UK; ^8^ 55980 Department of Neuropathology Royal Victoria Hospital Belfast Northern Ireland; ^9^ 156552 Department of Neurology Belfast City Hospital Belfast BT9 7AB UK; ^10^ 156552 Department of Genetic Medicine Belfast City Hospital Belfast BT9 7AB UK; ^11^ Neuromuscular and Rare Diseases Unit Department of Neuroscience IRCCS Foundation Ca’ Granda Ospedale Maggiore Policlinico Dino Ferrari Centre University of Milan Milan Italy; ^12^ Neuromuscular Diseases and Neuroimmunology Unit Fondazione IRCCS Isitituto Neurologico C. Besta Milano Italy; ^13^ 6396 Neurosciences Group Nuffield Department of Clinical Neurosciences Weatherall Institute of Molecular Medicine University of Oxford Oxford UK; ^14^ 61554 Neurogenetics Laboratory UCL Queen Square Institute of Neurology Queen Square WC1N 3BG London UK

## Abstract

**Objective:**

A group of genes have been reported to be associated with myopathies with tubular aggregates (TAs). Many cases with TAs still lack of genetic clarification. This study aims to explore the genetic background of cases with TAs in order to improve our knowledge of the pathogenesis of these rare pathological structures.

**Methods:**

Thirty‐three patients including two family members with biopsy confirmed TAs were collected. Whole‐exome sequencing was performed on 31 unrelated index patients and a candidate gene search strategy was conducted. The identified variants were confirmed by Sanger sequencing. The wild‐type and the mutant p.Ala11Thr of *ALG14* were transfected into human embryonic kidney 293 cells (HEK293), and western blot analysis was performed to quantify protein expression levels.

**Results:**

Eleven index cases (33%) were found to have pathogenic variant or likely pathogenic variants in *STIM1*, *ORAI1*, *PGAM2*, *SCN4A*, *CASQ1* and *ALG14*. Among them, the c.764A>T (p.Glu255Val) in *STIM1* and the c.1333G>C (p.Val445Leu) in *SCN4A* were novel. Western blot analysis showed that the expression of ALG14 protein was severely reduced in the mutant *ALG14* HEK293 cells (p.Ala11Thr) compared with wild type. The *ALG14* variants might be associated with TAs in patients with complex multisystem disorders.

**Interpretation:**

This study expands the phenotypic and genotypic spectrums of myopathies with TAs. Our findings further confirm previous hypothesis that genes related with calcium signalling pathway and N‐linked glycosylation pathway are the main genetic causes of myopathies with TAs.

## Introduction

Tubular aggregates (TAs), which are unusual membranous structures in skeletal muscle, were first described in 1964.^1^ They are found predominantly in type II muscle fibres, although they have been described in both type I and type II fibres.^2^ By enzyme histochemistry, TAs appear bright red with modified Gomori trichrome (mGT), and stain with periodic acid‐Schiff (PAS). They also react for myoadenylate deaminase (MAD), even without substrate, non‐specific esterase and nicotinamide adenine dinucleotide‐tetrazolium reductase (NADH‐TR). They do not react for the mitochondrial oxidative enzymes succinate dehydrogenase (SDH) or cytochrome c oxidase (COX).^3^ It is now well established that these structures originate from sarcoplasmic reticulum (SR) containing a number of different SR components.^4^ Ultrastructurally, TAs are characterised by the abnormal accumulation of densely packed tubules of variable forms and sizes located in skeletal muscle fibres.^5^

TAs are present in a wide variety of different disorders, which may occur either sporadically or in a hereditary manner.^1^, ^6^, ^7^, ^8^, ^9^ The major clinical symptoms of myopathies with TAs include exertional myalgia, muscle cramps and stiffness with or without weakness, slowly progressive proximal weakness, fatigability and periodic paralysis. Nine genes have been reported to be associated with cases with TAs. These genes are mainly associated with four clinical phenotypes––dominantly inherited tubular aggregate myopathy (TAM),^10^, ^11^ phosphoglycerate mutase deficiency (glycogenosis type X),^7^ congenital myasthenic syndromes^12^, ^13^, ^14^ and periodic paralysis^15^ (Table 1).

**Table 1:** Known genes associated with myopathies with tubular aggregates.

Gene ID	OMIM^a^	Clinical phenotype (OMIM)	Inheritance
***STIM1***	605921	Tubular aggregate myopathy (TAM) (160565) Stormorken syndrome (185070)	Autosomal dominant
***ORAI1***	610277	Tubular aggregate myopathy (160565) Stormorken syndrome (185070)	Autosomal dominant
***CASQ1***	114250	Myopathy, vacuolar, with CASQ1 aggregates (616231)	Autosomal dominant
***GFPT1***	138292	Congenital myasthenia, 12 (610542)	Autosomal recessive
***DPAGT1***	191350	Congenital myasthenia, 13 (614750)	Autosomal recessive
***ALG2***	607905	Congenital myasthenia, 14 (616228)	Autosomal recessive
***PGAM2***	612931	Glycogen storage disease X/ phosphoglycerate mutase deficiency (261670)	Autosomal recessive
***SCN4A***	603967	Hypokalemic periodic paralysis, type 2 (613345) Paralysis periodica paramyotonia	Autosomal dominant
***SERAC1***	614725	3‐methylglutaconic aciduria with deafness, encephalopathy and Leigh‐like syndrome (614739)	Autosomal recessive

^a^ OMIM = Online Mendelian Inheritance in Man

Among them, stromal interaction molecule 1 (STIM1) is a calcium sensing protein in the SR membrane, which binds to ORAI1, a calcium release‐activated channel (CRAC), mediating store‐operated calcium entry in the plasma membrane. *CASQ1* encoding calsequestrin 1 is a high‐capacity and moderate‐affinity calcium‐binding protein predominantly located at terminal cisternae and expressed in fast skeletal muscle fibres. It acts as the main calcium buffer of the SR.^16^ Proteins encoded by the *GFPT1* (glutamine‐fructose‐6‐phosphate transaminase 1), *DPAGT1* (dolichyl‐phosphate N‐acetyl‐glucosaminephosphotransferase 1) and *ALG2* (alpha‐1,3/1,6‐mannosyltransferase) genes, are all involved in the early steps of N‐linked (asparagine‐linked) protein glycosylation. Pathogenic variants in these genes are suggested to lead to reduced levels of acetylcholine receptors at the endplate region due to defects in glycosylation of acetylcholine receptor subunits.^14^, ^17^ Therefore, these suggested that the calcium dyshomeostasis and impaired N‐linked glycosylation play important role in the formation of TAs.^18^, ^19^

However, many cases with TAs still lack of genetic clarification. This study aims to explore the genetic background of cases with TAs in order to improve our knowledge of the pathogenesis of these rare pathological structures.

## Methods

**Figure 1:**
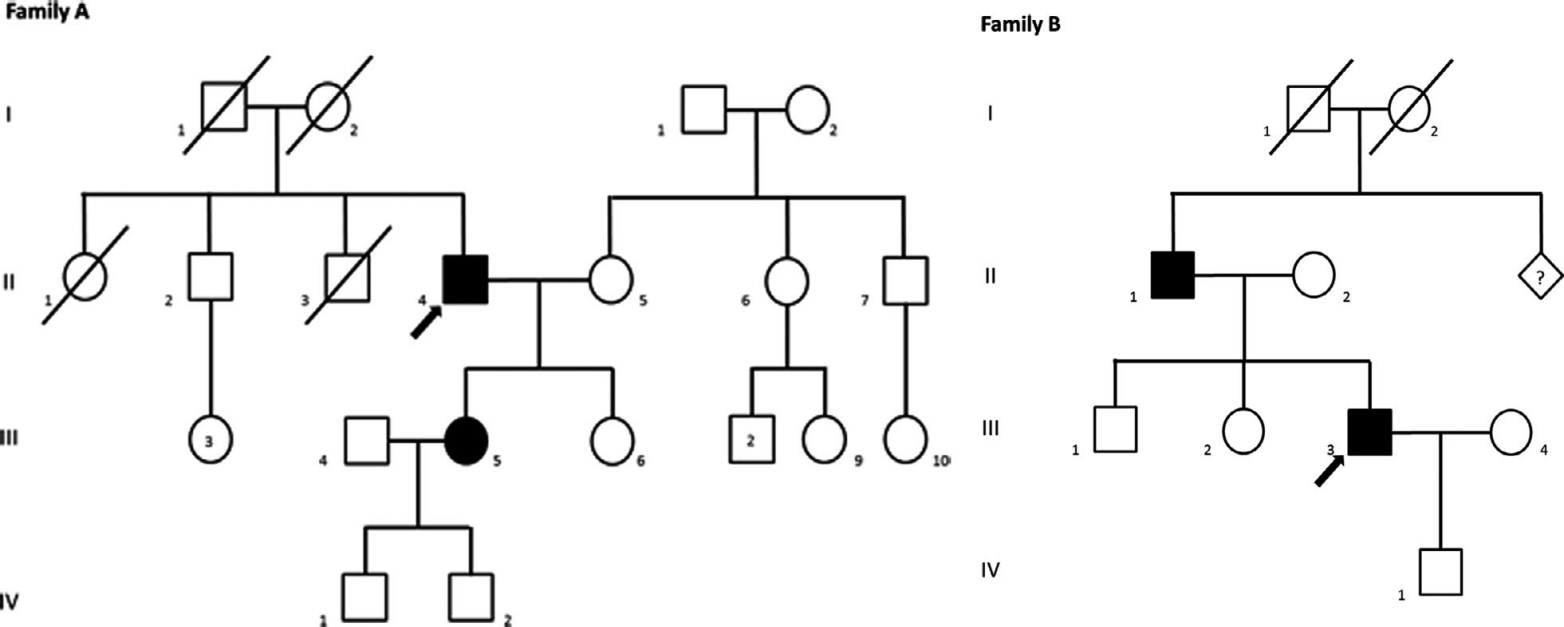
Pedigrees of the Family A and Family B. Black symbols represent affected patients; Black arrow points the index patient of the family; Symbols with slash indicate deceased individuals; Numbers inside the symbols indicate multiple individuals with the same gender; Diamond with a question mark indicates that the number of individuals with unknown gender was unclear.

A total of 33 biopsy confirmed with TAs but genetically undiagnosed patients were collected from September 2012 to January 2016. Twenty‐one cases were identified in the National Hospital for Neurology and Neurosurgery (NHNN), University College London (UCL). Four cases from two families (Figure 1 Families A and B) were from Belfast City Hospital in Northern Ireland, four unrelated cases were from the Neuromuscular Unit, BioBank of Skeletal Muscle, Nerve Tissue, DNA and Cell Lines in Italy and five were from Neuromuscular Diseases and Neuroimmunology Unit Muscle Cell Biology Lab in Italy. DNAs from blood or muscle samples were collected. Whole‐exome sequencing was performed on 31 unrelated index patients with TAs at UCL Queen Square Institute of Neurology.

The sequencing was carried out in‐house on the Illumina HiSeq2500 platform and an in‐house bioinformatics analysis pipeline was applied as previously described.^20^ A candidate gene searching strategy was performed. Variants identified in these genes were evaluated through protein expression studies. The detailed methods are available in the supplement.

Muscle biopsy slides were available for patients from the NHNN. Biopsies were reviewed and images were provided by the Division of Neuropathology, UCL Queen Square Institute of Neurology.

Written consent forms were obtained from all the patients according to the Declaration of Helsinki. The study was approved by the National Research Ethics Service (NRES) Committee London – Queen Square (REC reference: 12/LO/1557).

## Results

In this cohort, 31 cases (31/33, 94%) were males and two were females. Clinical data of five cases were missing. The age of onset ranged from childhood to 63 years old. Among them, 12 cases had a positive family history, 13 were sporadic and eight had unknown family history. Myalgia (14/33, 42%) and limb weakness (13/33, 39%) were the most frequent symptoms. Seven presented with muscle cramps (21%), five with fatigability (15%) and four with muscle stiffness (12%). In addition, six patients present with eye problems (18%) including tonic pupils, ophthalmoplegia, ptosis and intermittent diplopia, three patients with involuntary movements (9%) including myoclonic jerks, chorea and dystonia, three with swallowing difficulty (9%) and one with myoglobinuria (3%). For those patients with creatine kinase (CK) levels available, eight (8/11, 73%) had elevated CK levels. Among the five patients with available electromyography (EMG) and nerve conduction study (NCS) data, three showed myopathic changes, one had sensory and motor axonal polyneuropathy and one was normal. The clinical features of patient identified with genetic variants are listed in Supplementary Table S1.

Eleven index cases (33%) were found to have pathogenic or likely pathogenic variants in five known genes and one candidate gene, including *STIM1*, *ORAI1*, *PGAM2*, *SCN4A*, *CASQ1* and *ALG14*. Among them, two variants were not reported previously in public databases (Table 2).

**Table 2:** Genetic variants identified in known/candidate genes in this cohort.

Patient	Phenotype	Gene	Mutation	Clinical significance
A‐II‐4	Limb‐girdle muscular dystrophy	*STIM1*	p. Asp84Glu (het^a^)	Pathogenic
A‐III‐5	Exercise‐induced myalgia			
B‐III‐3	Congenital TAM^bc^	*STIM1*	**p. Glu255Val (het)**	Likely pathogenic
B‐II‐1	TAM			
Case 1	TAM	*STIM1*	p. Leu92Val (het)	Pathogenic
Case 2	Muscle weakness, fatigue, myalgia	*ORAI1*	p. Val107Met (het)	Pathogenic
Case 3	Exercise‐induced myalgia, myoglobinuria	*PGAM2*	p. Arg10Gln (hom^b^)	Pathogenic
Case 4	Myalgia, muscle cramps	*PGAM2*	p. Gly178fs30Ter (hom)	Pathogenic
Case 5	Myotonic syndrome	*SCN4A*	**p. Val445Leu (het)**	Likely pathogenic
Case 6	Exercise‐induced muscle spasms and cramps	*CASQ1*	p. Asp44Asn (het)	Pathogenic
Case 7	Epilepsy, hearing loss, facial weakness	*ALG14*	p. Arg104Ter (het)	Pathogenic
			p. Ala11Thr (het)	Likely pathogenic

^a^ het = heterozygous; ^b^hom = homozygous

^c^ TAM = tubular aggregate myopathy; novel mutation labelled in Bold

### Family A

A missense variant c.252T>A (p. Asp84Glu) in the *STIM1* gene was found in Family A‐II‐4 and segregated in his affected daughter (A‐III‐5) (Figure 1). This variant has been previously reported to be pathogenic in TAM.^21^ The phenotypes of Family A were previously published in detail by Cameron et al.^2^ Their age of onset was from childhood. Both patients present with progressive muscle weakness. A‐III‐5 also present with muscle pain after exercise. Histochemical staining of muscle biopsy showed features of TAs in the index patient, while that of the daughter showed relatively normal fibres with occasional central nuclei. Under electron microscope (EM), there was a similar ultrastructural appearance in both patients. Large TAs were predominant in all fibres in the index patient, while they were much smaller in the daughter. The p.Asp84Glu variant was confirmed to be segregating in A‐III‐5.

### Family B

Family B‐III‐3 (Figure 1) was found to have a missense variant c.764A>T (p.Glu255Val) which is a novel variant absent from the 1000 Genomes project, EVS and gnomAD databases and is predicted as pathogenic by SIFT, PolyPhen2 and MutationTaster. This variant was confirmed in B‐II‐1 and was segregated with the disease in this family. Both the index case (B‐III‐3) and his father (B‐II‐1) presented with same phenotype of muscle weakness and ophthalmoplegia.

### Case 1

Case 1 was found to have a missense variant c.274C>G (p.Leu92Val) in the *STIM1* gene. This variant has also been previously reported to be pathogenic in TAM.^22^ The patient developed muscle weakness in the lower limbs and myalgia from the age of 15. She had elevated CK levels at 2000 IU/L, and a normal EMG. However, her family history was unknown.

### Case 2

**Figure 2:**
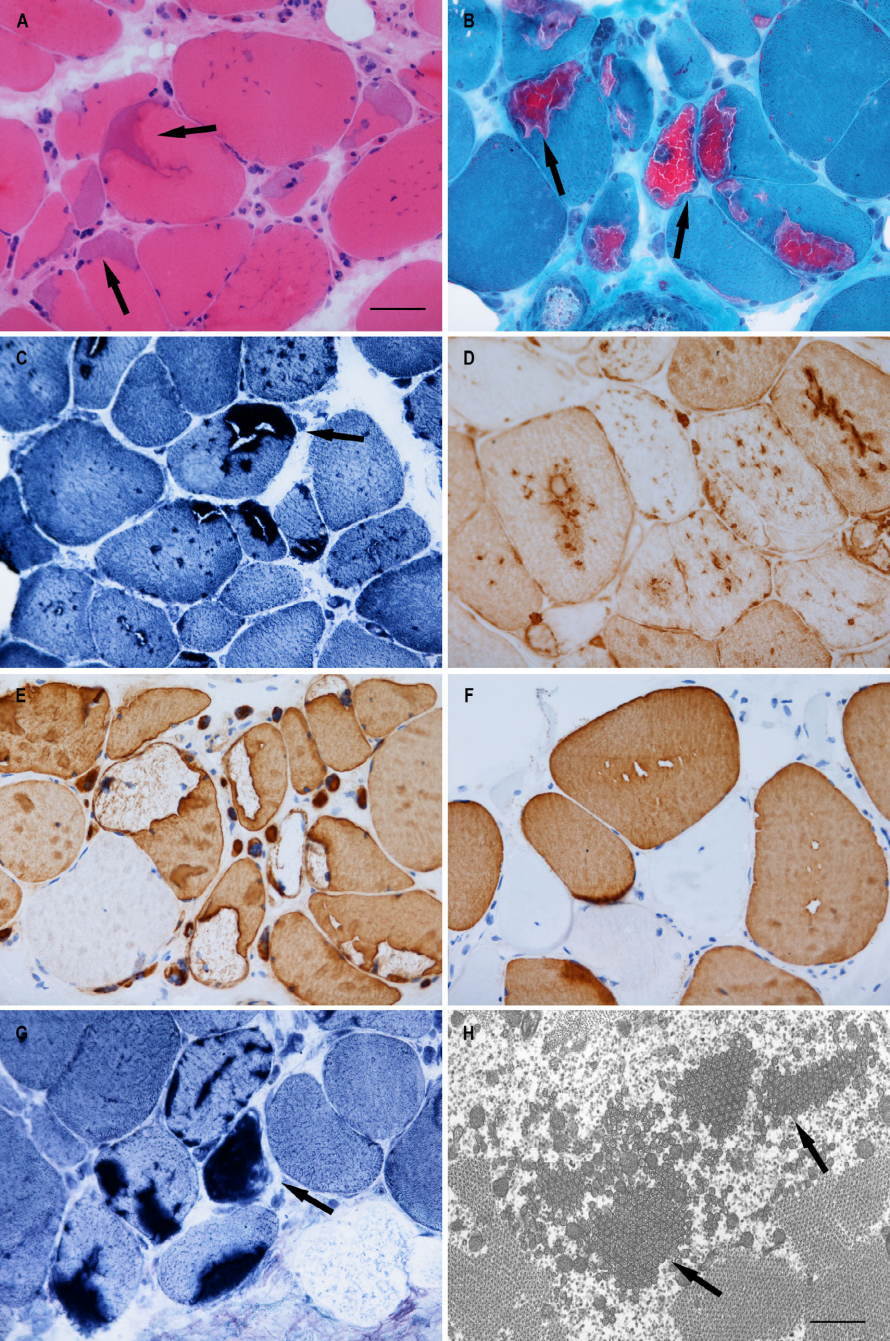
Pathological changes observed in Case 2. Hematoxylin and eosin staining shows variation in fibre size with frequent atrophic fibres and increased connective tissue. Prominent basophilic inclusions are present in many fibres (A); Modified Gomori trichrome staining highlights bright red inclusions (B); The inclusions were stained dark in NADH‐TR (C) and adenylate deaminase preparations (G); The inclusions were immunoreactive for SERCA II (D); Fast myosin (E) and slow myosin (F) immunohistochemistry indicated both type I and type II fibres were affected; Accumulations of double‐walled tubular aggregates were confirmed in electron microscopy (EM) (H). Inclusions of tubular aggregates are indicated by arrows in images A‐C, G and H. Scale bar represents 50 μm in A‐G for histology images; Scale bar in H represents 500 nm in EM

A missense variant c.319G>A (p.Val107Met) in the *ORAI1* gene was identified in Case 2. This variant has been previously reported to be pathogenic in a family with TAM.^23^ Detailed clinical features of Case 2 were previously described as a case report.^24^ This patient started experiencing symptoms of upper limb weakness and fatigue, and myalgia in the lower limbs from the age of 47. A muscle biopsy from Case 2 was performed at the age of 50 and it showed multiple accumulations of TAs in both type I and type II fibres (Figure 2A‐G). EM confirmed the presence of accumulated double‐walled TAs (Figure 2H).

### Cases 3 and 4

**Figure 3:**
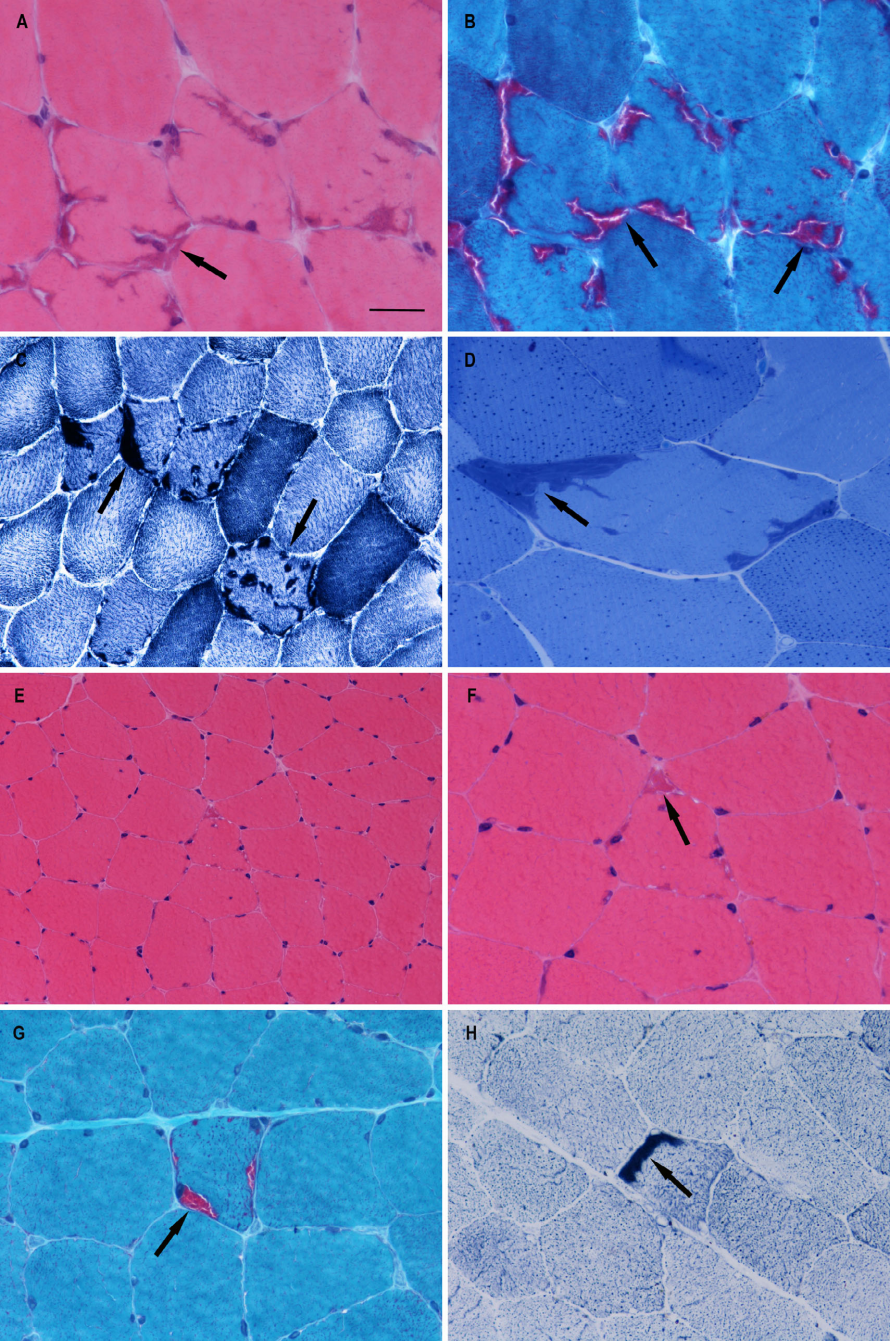
Pathological changes observed in Case 3 and Case 5. Case 3 (A‐D): There were numerous subsarcolemmal and internal basophilic inclusions suggesting TAs in muscle fibres stained using hematoxylin and eosin (A), while these inclusions were stained red in the modified Gomori trichrome preparation (B), and were stained as dark in NADH‐TR and mainly in type II fibres (C); In toluidine blue, the inclusions were in dark blue, with small dark dots in fibres representing lipid droplets (D); Case 5 (E‐H): There was increased variation in fibre size and scattered atrophic fibres which were mostly angular or polygonal in outline. Occasional fibres contain basophilic inclusions suggestive of TAs when stained with hematoxylin and eosin (E, F); TAs are stained red in the modified Gomori trichrome preparation (G), and are darkly stained in NADH‐TR (H). Inclusions of TAs are indicated by arrows in all the images. Scale bar represents 25 μm in A, B, D and F‐H; represents 50 μm in C and E.

Two patients were found to harbour pathogenic variants in the *PGAM2* gene. A homozygous missense variant c.29G>A (p. Arg10Gln) was identified in Case 3, and a homozygous frameshift deletion c.532delG (p.Gly178fs30Ter) in Case 4. The p.Arg10Gln was previously reported in a patient with persistent elevation of serum CK, but without TAs.^25^ The p.Gly178fs30Ter deletion is a known mutation in a patient with PGAM deficiency. The deletion causes a frameshift in exon 2 resulting in a premature termination codon.^26^ Both Case 3 and Case 4 were from consanguineous families. They both presented with myalgia and muscle cramps, and elevated serum CK levels. Case 3 additionally had episodes of myoglobinuria. Muscle biopsy from Case 3 showed numerous type II muscle fibres with TAs which were often subsarcolemmal but also internal (Figure 3A‐D). Both the phosphorylase and adenylate deaminase activities were increased in TAs, which also had increased glycogen content.

### Case 5

A heterozygous missense variant in exon 9 c.1333G>C (p.Val445Leu) of *SCN4A* was identified in Case 5. This is predicted as probably deleterious by SIFT, PolyPhen2 and MutationTaster. The same amino acid substitution p.Val445Leu but with a different nucleotide c.1333G>T was reported in a Chinese patient with sodium channel myotonia.^27^ Our patient first developed symptoms around the age of 12. His muscles became very stiff and painful after rugby trainings, so that he was unable to walk. The stiffness mostly affected his legs but occasionally also in his hands, abdominal muscles and the eyelids. CK levels were mildly elevated around 204‐216 IU/L. EMG showed significant length‐dependent sensory motor polyneuropathy with axonal features, but no myotonic discharges. Muscle biopsy at the age of 59 revealed features suggestive of acute denervation, and a small number of fibres with TAs (Figure 3E‐H). His father and two children had symptoms of muscle stiffness, which suggests an autosomal dominant form of inheritance. But DNA samples of other families were not available for segregation analysis.

### Case 6

A heterozygous missense variant in *CASQ1* (c.G130A, p.Asp44Asn) was found in Case 6. This variant has been previously reported in a family with TAM who complained of fatigue and diffuse exercise‐induced myalgia.^28^ The patient had exercise‐induced muscle spasms since childhood. His mother also had similar muscle cramps, suggesting an autosomal dominant form of inheritance. However, DNA from his mother was not available for segregation analysis.

### Case 7

**Figure 4:**
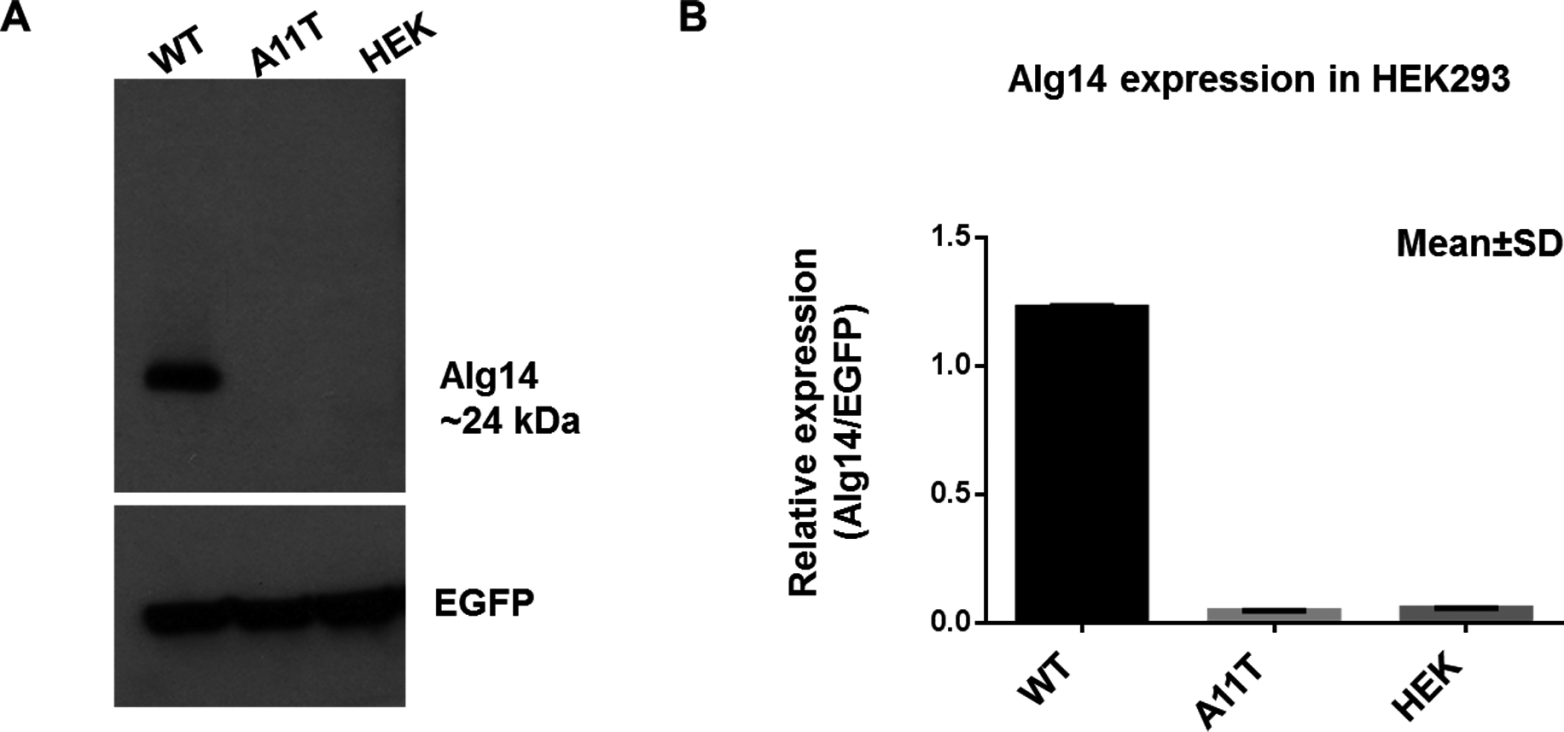
Functional analysis of the variant p. Ala11Thr in the *ALG14* gene. (A) Western blot of HEK293 cells with wild type and p. Ala11Thr mutant ALG14. Transfection efficiency was verified by co‐transfection of EGFP. (B) The level of ALG14 expression was the mean value of three experiments, and the error bar represents the standard deviation. EGFP, enhanced green fluorescent protein; HEK, human embryonic kidney 293; WT, wild type.

Compound heterozygous stop‐gain mutation c.310C>T (p.Arg104Ter) and missense variant c.31G>A (p.Ala11Thr) in a candidate gene *ALG14* were found in Case 7. The patient first developed symptoms in his early 20s. The main manifestations were generalised tonic‐clonic seizures, impaired hearing and bilateral facial weakness. His electroencephalogram (EEG) showed the moderate widespread abnormalities of background activity with independent epileptiform abnormalities over right frontotemporal and left temporal regions. His EMG was normal. His two family members also had hearing difficulties, but their detailed clinical symptoms were not available. The heterozygous variant c.310C>T was previously reported to be pathogenic in a family of two siblings with a diagnosis of probable congenital myasthenic syndrome and without TAs in muscle biopsies.^14^ The variant introduces a premature stop codon after amino acid residue Arg104, resulting in a truncated protein by 112 amino acid residues. A heterozygous variant p.Ala11Thr was previously reported in a patient with foetal alcohol syndrome, but its functional effect was unknown.^29^ Functional analysis showed that the expression of the ALG14 protein was severely reduced in the HEK293 cells with homozygous mutant Ala11Thr compared with the wild type. This suggests that the p.Ala11Thr variant is pathogenic in a loss‐of‐function manner (Figure 4).

## Discussion

In this cohort, we identified seven patients with pathogenic/likely pathogenic rare variants in three calcium homoeostasis‐related genes (*STIM1*, *ORAI1* and *CASQ1*), and two patients with pathogenic rare variants in the *PGAM2* gene, one with the *SCN4A* gene and one with an N‐linked glycosylation‐related gene (*ALG14*). The success rate of genetic diagnosis in this cohort is 33%. These genetic findings provide further evidence for the previous hypothesis that disordered calcium homoeostasis and impaired N‐linked glycosylation pathways are involved in the formation of the TAs.

### TAs associated with dysregulation of calcium homoeostasis

Both p.Asp84Glu and p.Leu92Val lie in the EF‐hand domain, which is known to be a mutation hotspot in the *STIM1* gene and causes dominant TAM through a gain‐of‐function mechanism. The p.Asp84Glu was previously reported in a case with TAM and additional features of Stormorken syndrome such as asplenia and short stature. Functional analysis also showed that this mutation triggered STIM1 clustering independent of calcium store depletion.^21^ This suggested that the mutant STIM1 was unable to sense calcium in the SR lumen thereby being oligomerised and clustered at the SR plasma‐membrane junctions to activate CRACs. Therefore, the p.Leu92Val is postulated to underlie the similar pathogenic mechanism to induce the phenotypes of the disease.

The p.Glu255Val is a novel variant which is located in the cytoplasmic coiled‐coil 1 (CC1) domain of STIM1. In the inactive state, CC1 domain binds to CRAC‐activating domain (CAD) / STIM1‐Orai‐activating region (SOAR) forming a folded STIM1 C‐terminus. When STIM1 is activated by the depletion of SR calcium, the conformation of CC1‐CAD/SOAR is extended. This is a critical process in binding to ORAI1 in the plasma membrane and to activate the CRAC channel.^30^ Mutations in this region may affect the interaction between CC1 and CAD/SOAR in the inactive state. Further analysis would be required to understand the exact consequence of the p.Glu255Val.

ORAI1 is a tetra‐spanning transmembrane protein in the plasma membrane, and acts as an ion‐conducting pore subunit of the CRAC channel. ORAI1 consists of four alpha‐helical transmembrane domains (M1‐M4), two extracellular loops and intracellular N‐ and C‐terminus. The functional CRAC channel is a hexamer of ORAI1 proteins, of which the central ion pore is composed of a ring of six M1 domains. Amino acid residues in the M1 domain are thought to have functions in both channel activity and the ion selectivity of store‐operated calcium entry (SOCE) channels. The p.Val107Met affects an amino acid in the M1 domain through a gain‐of‐function mechanism.

The interaction of ORAI1 and STIM1 proteins activates the opening of CRAC and SOCE channels, which plays an essential role in calcium signalling pathway resulting in calcium influx into the cytosol.^30^ A recent study summarised that gain‐of‐function mutations in *STIM1* and *ORAI1* genes were associated with TAM and Stormorken syndrome (TAM/STRMK), in contrast, loss‐of‐function mutations were associated with CRAC channelopathy involving immunodeficiency and autoimmunity, muscle hypotonia, ectodermal dysplasia and mydriasis.^31^ TAM/STRMK and CRAC channelopathy patients present mirror phenotypes in accordance with the opposite pathomechanisms.^31^

All of our *STIM1* patients presented mainly with symptoms of muscle weakness and myalgia with elevated CK levels, and there was no evidence suggestive of Stormorken syndrome. Both Family A and Family B presented with ophthalmoparesis, which has been reported in eight other patients.^30^ The two affected patients in Family A also had severe lordosis, which has also been described in previous cases.^32^, ^33^ Case 2 with the *ORAI1* gene present with not only muscle weakness, stiffness and myalgia, but also a mild saddle nose deformity, pectus excavatum and miosis. This may suggest a diagnosis of TAM/STRMK.

The variant p.Asp44Asn in the *CASQ1* gene found in Case 6 has been reported previously in a patient with TAM. Functional analysis showed that a reduced calcium‐dependent aggregation for the p.Asp44Asn mutant CASQ1, and a reduced ability to store calcium and a reduced inhibitory effect on SOCE.^28^

TAs originate from the SR. SR‐related proteins involved in the uptake and storage of calcium, such as CASQ1, RYR1 and SERCA, have been observed to be components of TAs.^34^ It is suggested that dysregulated intraluminal or cytosolic calcium level could potentially cause the swelling of SR cisternae and their extension into longitudinally oriented tubules, subsequently resulting in the formation of TAs.^35^ A similar process was also proposed for the sequential formation TAs in ageing skeletal muscle fibres.^36^

### TAs associated with the impaired N‐linked glycosylation pathway

Another major group of genetic factors related to TAs are recessive mutations in genes encoding proteins involved in N‐linked glycosylation pathway, including previously reported *GFPT1*, *DPAGT1* and *ALG2*, and *ALG14* found in our cohort. Protein N‐linked glycosylation plays a critical role in a wide variety of biological processes, such as protein folding, cellular targeting and motility and immune response.^37^ Both STIM1 and ORAI1 are glycosylated proteins. A study reported that a mutation at N‐glycosylation site in STIM1 resulted in a strong gain‐of‐function effect by increasing the number of active CRAC channels. This finding showed the importance of N‐glycosyl modification for the function of STIM1.^38^ Thus, it is possible that mutations in genes involved in the N‐linked glycosylation pathway may cause inappropriate glycosylation of STIM1 and other proteins. This leads to abnormal protein folding and impaired calcium homoeostasis, and eventually triggers the proliferation of SR tubules and formation of TAs. Further analyses of CRAC channel activation in skeletal muscles of patients with mutations in this group of genes would be required in the future.

*ALG14*, similarly to *ALG2* and *DPAGT1*, encodes a protein involved in the early steps of N‐linked glycosylation. Studies in yeast have shown that ALG14 is a membrane protein and plays an essential role in recruiting ALG13 to the endoplasmic reticulum and form a heterodimer. It catalyses the first two steps of the biosynthesis of lipid‐linked oligosaccharide (LLO) precursor for N‐glycan assembly. Mutations in *ALG14* have been related to the phenotypes of CMS,^14^ and also congenital disorders of glycosylation (CDG). CDG are known as complex multisystem disorders with symptoms including cerebral atrophy, therapy‐refractory epilepsy, severe intellectual disability, behavioural problems and mild dysmorphic features.^39^ The variant p.Arg104Ter in *ALG14* was known to be pathogenic. Although the p.Ala11Thr variant is predicted as benign in silico, our functional analysis showed that the p.Ala11Thr variant markedly reduced the expression of ALG14. Case 7 presented with generalized seizures, hearing loss, bilateral facial weakness, intellectual disability and arachnodactyly and Marfanoid appearance which is likely due to the combination of two deleterious variants in *ALG14* leading to a complex multisystem disorder. However, we cannot rule out the possibility that the two variants are in cis due to the inability of performing the segregation analysis. Our finding suggests that mutations in the *ALG14* gene might be associated with TAs, but replication studies would be important to clarify the effect of these variants. It would be worthwhile to examine the muscle tissue of patients with variants in the *ALG14* gene in the future.

### TAs are unspecific pathological changes in muscle

The *PGAM2* gene encodes muscle phosphoglycerate mutase‐2 which is a glycolytic enzyme that catalyses the interconversion of 2‐phosphoglycerate and 3‐phosphoglycerate using 2,3‐bisphosphoglycerate as a cofactor.^40^ Mutations in *PGAM2* are known to cause PGAM deficiency. Case 3 with homozygous p.Arg10Gln substitution and Case 4 with homozygous p.Gly178fs30Ter mutation had the typical clinical presentation of PGAM deficiency since they both had myalgia and muscle cramps, and elevated serum CK levels. PGAM activities were found reduced to 3% and 5% of normal, respectively in two previous cases with the variant p.Arg10Gln and deletion p.Gly178fs30Ter.^25^, ^26^ Case 4 had symptom onset from infancy, which indicates that the frameshift deletion may cause a more severe phenotype compared with the missense mutations. The previous case with homozygous p.Gly178fs30Ter had late onset of symptoms, and was additionally diagnosed with a possible late‐onset statin myopathy. The authors suggested that clinically silent metabolic myopathies might be unmasked due to the exposure to statins.^26^

The variant c.1333G>C in the *SCN4A* gene causes the same amino acid substitution p.Val445Leu as the variant c.1333G>T which was reported in a Chinese patient with sodium channel myotonia. The p.Val445Leu variant is located in the sixth transmembrane segment of domain 1 of the skeletal muscle sodium channel (*SCN4A*). Despite the segregation in other members of the family could not be performed, we consider that p.Val445Leu variant is likely to cause the patient’s myotonic phenotype, based on the likely autosomal dominant inheritance of this family, and the clinical phenotypes of the patient. However, this patient did not present with myotonic discharges in EMG which was different from the previous case.

The pathogenesis of *PGAM2*‐ and *SCN4A*‐related TAs is still a mystery. However, interestingly, two patients with PGAM deficiency and TAs were observed with markedly increased calcium concentration and calcium adenine triphosphate activity in muscles.^26^ It is speculated that abnormal calcium homoeostasis is likely to be a common final process leading to the formation of TAs, which is induced by diverse conditions. These may include defects in calcium signalling pathway and N‐linked glycosylation pathway, PGAM deficiency, probably also exposure to drugs, toxins and hypoxia. Meanwhile, there may be other mechanisms underlying the pathogenesis of TAs given that in two thirds of the cases in our cohort we did not identify rare genetic variants in the pathways discussed above.

It is also interesting to see that TAs associated with calcium homoeostasis genes are larger and more dominant in muscle fibres compared with those associated with other genes, such as *PGAM2* and *SCN4A*. This may also suggest that direct impairment in calcium homoeostasis pathway could cause more severe damage in muscle. However, all these data further confirm that TAs are a gradually changing SR architecture, and also an unspecific pathological change as an adaptive response of the SR to various insults to the muscle fibres.

## Conclusion

WES was applied in a cohort of patients with myopathies with TAs and the results from this study expanded the phenotypic and genotypic spectrums of myopathies with TAs. Our study confirms previous hypothesis that genes related with calcium signalling pathway and N‐linked glycosylation pathway are the main genetic causes of myopathies with TAs so far. Due to genetic causes have yet been identified in 67% of our cases, whole‐genome sequencing could be applied in the future to explore other potential genetic causes of TAs. TAs are also non‐specific pathological features secondarily responding to different conditions.

## Author Contributions

Q.G. contributed to all the experimental work, data analysis and drafting the first version of the paper. J.L.H. contributed to the review and photography of the muscle biopsies. WW.L. performed the western blots. All authors contributed to the revision of the manuscript.

## Conflict of Interests

All authors have no competing financial interests.

## Supporting information


**Table S1**. Clinical features of 11 cases identified with known/candidate genes.
**Table S2**. Genetic mutations/variants identified in known/candidate genes in this cohort.
**Supplementary Methods**. Methods for genetic analysis and functional analysis.

## Data Availability

Any data not published with the article will be shared by request from any qualified investigator.
