# Supplementary material for: Genetic defects are common in myopathies with tubular aggregates
Source: Ann Clin Transl Neurol. 2021 Dec 15;9(1):4–15. doi: 10.1002/acn3.51477 (PMC8791796; doi:10.1002/acn3.51477)
Supplement: Supplementary file 1 — Table S1. Clinical features of 11 cases identified with known/candidate genes. Table S2. Genetic mutations/variants identified in known/candidate genes in this cohort. Supplementary Methods. Methods for genetic analysis and functional analysis. [file ACN3-9-4-s001.docx]

**Supplementary Table 1 Clinical features of 11 cases identified with known/candidate genes**

| Case ID | Sex | AAO | FxH | Clinical Manifestations | CK level | EMG/NCS | Muscle Biopsy |
| --- | --- | --- | --- | --- | --- | --- | --- |
| A-II-4 (index) | M | Early childhood | Y | (1) Limb-girdle muscular dystrophy;  (2) Lordosis, walk with a waddling gait;  (3) Bilateral restriction of eye adduction and upward gaze | 1550 IU/L | EMG: Increase in polyphasic activity in motor unit potentials | IHC: features of TAs;  EM: confirmed TAs; large TAs predominantly in all fibres types |
| A-III-5 | F | About nine | Y | (1) Progressive muscle weakness, and muscle pain after exercise;  (2) Lordosis, walk with a waddling gait;  (3) Ophthalmoparesis with mostly up-gaze and some horizontal restrictions | 700 IU/L | EMG: a predominantly myopathic pattern;  NCS: within normal limits | IHC: relatively normal fibres with increased  EM: confirmed TAs; TAs much smaller compared with above |
| B-III-3 (index) | M | UK | Y | (1) Congenital myopathy with tubular aggregates;  (2) Ophthalmoparesis | N/A | N/A | EM confirmed TAs |
| B-II-1 | M | UK | Y | TAM | N/A | N/A | EM confirmed TAs |
| Case 1 | M | 15y | UK | (1) TAM;  (2) Muscle weakness in lower limbs and myalgia | 2000 IU/L | N/A | EM confirmed TAs |
| Case 2 | M | 47y | N | (1) Muscle weakness and fatigue in the upper limbs and myalgia in the lower limbs;  (2) A mild saddle nose deformity and pectus excavatum;  (3) tonic pupils | 376-3854 IU/L | Mild chronic myopathic but not neuropathic changes | Figure 2 |
| Case 3 | M | 26y | Consanguineous parents | (1) Myalgia and anuria after intense exercise; (2) Myoglobinuria | > 2000 IU/L | N/A | IHC:  Figure 3 A-D |
| Case 4 | M | A few months | Consanguineous parents | Myalgia and muscle cramps | 606-76000 IU/L | EMG: myopathic pattern | IHC:  presence of subsarcolemmal TAs in over 50% of fibres with PAS-positive vacuoles |
| Case 5 | M | 12y | Y | (1) Myotonic syndrome;  (2) Muscle stiffness and myalgia mostly in legs, occasionally in hands, abdominal muscle and eyelids | Mildly elevated around 204-216 IU/L | Length dependent sensory motor polyneuropathy with both axonal features and evidence of motor axonal loss. | IHC: acute denervation, and a small number of fibres with TAs (Figure 3 E-H) |
| Case 6 | M | Childhood | Y | (1) Muscle spasms in toes and arches of both feet;  (2) Symptoms triggered by exercise, and worsen in cold or wet weather;  (3) Jaw cramp when yawning | 527 IU/L | EMG: limited information as patient fainted;  NCS: normal | IHC: features of TAs confined to type II fibres;  EM: confirmed TAs |
| Case 7 | M | Early 20s | Y | (1) Generalised tonic-clonic seizures - these occurred once every one or three weeks;  (2) Inherited cochlear hearing loss;  (3) Bilateral facial weakness;  (4) Slightly dysarthrosis;  (5) Arachnodactyly and Marfanoid appearance;  (6) Mental retardation;  (7) Mild gait ataxia;  (8) Mild weakness of orbicularis oculi | Normal | Normal study without evidence of a large fibre neuropathy or myopathy | IHC: suggestive of TAs but no other abnormalities;  EM: about 5% of fibres with TAs; they were associated with areas of glycogen rich sarcoplasm; no evidence of mitochondrial abnormalities |

AAO = age at onset; FxH = family history; CK = creatine kinase; EMG = electromyograms; NCS = nerve conduction studies; F = female; M = male; IHC = immunohistochemistry; EM = electron microscopy; TAs = tubular aggregates; TAM = tubular aggregate myopathy; Y = yes; N/A = not available; UK = unknown

**Supplementary Table 2 Genetic mutations/variants identified in known/candidate genes in this cohort**

| Patient | Phenotype | Gene | Mutation | MAF in 1000G/EVS/ExAC | GERP++ | CADD | PolyPhen2/SIFT/MutationTaster |
| --- | --- | --- | --- | --- | --- | --- | --- |
| A-II-4 | Limb-girdle muscular dystrophy | *STIM1* | p.Asp84Glu (het) | N/A; N/A; N/A | 4.21 | N/A | D/T/D |
| A-III-5 | Exercise-induced myalgia |  |  |  |  |  |  |
| B-III-3 | Congenital TAM | *STIM1* | p.Glu255Val (het) | N/A; N/A; N/A | 5.38 | 4.98 | D/D/D |
| B-II-1 | TAM |  |  |  |  |  |  |
| Case 1 | TAM | *STIM1* | p.Leu92Val (het) | N/A; N/A; N/A | 2.55 | N/A | D/D/D |
| Case 2 | Muscle weakness, fatigue, myalgia | *ORAI1* | p.Val107Met (het) | N/A; N/A; N/A | 5.36 | 4.32 | D/D/D |
| Case 3 | Exercise-induced myalgia, myoglobinuria | *PGAM2* | p.Arg10Gln (hom) | N/A; N/A; 0.00004066 | 5.38 | 4.78 | D/D/D |
| Case 4 | Myalgia, muscle cramps | *PGAM2* | p.Gly178fs30Ter (hom) | N/A; 0.00008; 0.00005694 | N/A | N/A | N/A |
| Case 5 | Myotonic syndrome | *SCN4A* | p.Val445Leu (het) | N/A; N/A; N/A | N/A | 4.49 | D/D/D |
| Case 6 | Exercise-induced muscle spasms and cramps | *CASQ1* | p.Asp44Asn (het) | 0.002384; N/A; | 3.56 |  | D/D/D |
|  |  | *DPAGT1* | p.Gly251Glu (het) |  | 5.55 | 5.23 | D/D/D |
| Case 7 | Epilepsy, hearing loss, facial weakness | *ALG14* | p.Arg104Ter (het) | N/A; 0.000077; 0.00005774 | 5.39 | 7.34 | N/A/N/A/D |
|  |  |  | p.Ala11Thr (het) | 0.0018; 0.008227; 0.006945 | -0.309 | N/A | B/T/B |

MAF = minor allele frequency; 1000G = 1000 Genomes project; EVS = Exome Variants Server; ExAC = Exome Aggregation Consortium; GERP = Genomic Evolutionary Rate Profiling; CADD = Combined Annotation Dependent Depletion; het = heterozygous; N/A = not available; D = damaging

**Methods**

**Genetics analysis**

Genetic annotation was first screen for the non-synonymous rare variants with minor allele frequency (MAF) < 1% in 1000 Genomes project, Exome Variant Server and the Exome Aggregation Consortium (ExAC) databases among the reported neuromuscular genes downloaded from the online GeneTable of Neuromuscular Disorders (http://www.musclegenetable.fr). In addition, a candidate gene searching mainly focused on nine genes known to encode proteins related to sarcoplasmic reticulum (*RYR1*, *TRDN*, *ATP2A1*, *DHPR*, *SRL*), and proteins involved in N-linked protein glycosylation associated with congenital myasthenic syndromes (*ALP2*, *ALG13*, *ALG14*, *GMPPB*). Sanger sequencing was performed to confirm the variants identified in WES.

**Functional analysis**

HEK293 cells were seeded at 3x10^5^ per well in 6-well plates and transfected with 3 µg pDNA3.1/hygro(+) HA (human influenza hemagglutinin)-tagged wild type and mutant (Ala11Thr, A11T) Alg14 constructs using polyethyleneimine. Cells were harvested and lysed 48 hours after transfection. Whole cell lysates were subjected to western blotting. The expression of HA-tagged wild type and mutant Alg14 were detected using mouse monoclonal anti-HA antibody (at 1:1000 dilution, ab18181, Abcam), HRP (horseradish peroxidase)-conjugated anti-mouse secondary antibody (Dako) and ECL (GM Healthcare). Transfection efficiency was verified by co-transfection of EGFP (enhanced green fluorescent protein). The experiments were done in triplicate. Densitometry of bands was analysed using ImageJ software and the protein expression indicated as Alg14:EGFP was then quantitated.
